# The procurement of innovation by the U.S. government

**DOI:** 10.1371/journal.pone.0218927

**Published:** 2019-08-12

**Authors:** Gaétan de Rassenfosse, Adam Jaffe, Emilio Raiteri

**Affiliations:** 1 Chair of Innovation and IP Policy, College of Management of Technology, Ecole Polytechnique Fédérale de Lausanne, EPFL, Lausanne, Switzerland; 2 MIT Sloan School of Management, Cambridge, MA, United States of America; 3 Brandeis University, Waltham, MA, United States of America; 4 Motu Economic and Public Policy Research, Wellington, New Zealand; 5 Queensland University of Technology, Brisbane, Australia; 6 School of Innovation Sciences, Eindhoven University of Technology, Eindhoven, Netherlands; Indiana University Bloomington, UNITED STATES

## Abstract

The U.S. government invests more than $50 billion per year in R&D procurement but we know little about the outcomes of these investments. We have traced all the patents arising from government funding since the year 2000. About 1.5 percent of all R&D procurement contracts have led to at least one patent for a total of about 13,000 patents. However, contracts connected to patents account for 36 per cent of overall contract value. The gestation lag from the signing date of the contract to the patent filing is on average 33 months and does not depend on the type of R&D performed. Patents that are produced faster also seem to be more valuable. We find strong decreasing returns to contract size. Conditional on generating at least one patent, a 1-percent increase in the size of an R&D contract is associated with 0.12 percent more patents.

## Introduction

The U.S. government invests more than $50 billion per year in R&D procurement and about the same amount in research grants. These amounts represent about two-thirds of all total federal spending in R&D. (Intramural R&D carried out directly by the federal agencies accounts for the rest.) Federal agencies use procurement contracts when they seek to acquire products or services for their own benefit. Grants are instead preferred when an agency seeks to support a public purpose (31 U.S.C. § 6301-04). Despite the sheer size of spending, surprisingly little research has been conducted on government-sponsored R&D.

As government budget for science and innovation is put under pressure, the procurement of innovation deserves greater scrutiny. Broad-based understanding of the effects of this large expenditure on the innovation system requires broad-based information on the overall funding portfolio and its outputs. To facilitate this task, we have traced all the patents arising from procurement contracts and grants active between 2000 and 2013. We have identified the governmental agency and the procurement contract/grant number associated with each patent and matched these to administrative data in order to recover detailed contract and grant-level information. We have also searched for information on the scientific publications that are associated with these records in the form of funding acknowledgement.

The contribution of the present paper is twofold. First, it introduces the 3PFL database (Patents and Publications with a Public Funding Linkage), which contains information on both procurement contracts and research grants. Data are available on Zenodo. Second, it offers an empirical analysis of procurement contracts. The scope of the database differs from the scope of the analysis, owing to the fact that research grants have been studied elsewhere [1–8].

## Methods

To construct the 3PFL database we take advantage of the U.S. Federal Acquisition Regulation (FAR). The FAR regulates the federal procurement process and stipulates that federal contractors may retain title to inventions made in the performance of work under a Government contract. When the contractor decides to take title to an invention, it should timely file a patent application and grant the Government an irrevocable license to use the invention. To ensure that the government receives the license, the FAR requires the contractor to include in the U.S. patent document a statement acknowledging Government support and reporting information about the funding agency and the contract identification number. Regarding research grants, the Bayh-Dole Act imposes requirements similar to FAR for recipients of federally funded research grants. The grantee seeking patent protection for such inventions shall mention the grant number and the agency that issued the grant in the government interest statement. In September 2006, the U.S. Congress approved the Federal Funding Accountability and Transparency Act (FFATA). The Act requires federal contract, grant, loan, and other financial assistance awards to be displayed on a searchable, publicly accessible website in order to give the American public access to information on how tax dollars are being spent. The USAspending.gov website was launched in 2007 to comply with the FFATA’s requirements and provides abundant information about federal contracts and grants, including identification numbers. The FAR and FFATA requirements thus allow us to identify federally funded patents and to link them to the associated contract(s) and grant(s). We exploit these legal requirements to construct the 3PFL database.

The database construction involves four main steps. First, a Python script parses the full-text data of all patents granted by the U.S Patent and Trademark Office (USPTO) between 2005 and 2015. These data are stored in 52 weekly files per year, available in XML format. The script identifies the patents that contain a government interest statement and extracts contract and grant identifiers from the statement. Second, we match this information with the contract-level and grant-level information recovered from USAspending.gov exploiting the identification numbers. Third, we match the patent numbers with the European Patent Office’s worldwide statistical database (PATSTAT) to recover additional bibliographic information on the funded patents. Finally, by exploiting Clarivate’s Web of Science (WoS) database, we match the contracts from the identified patent-contract pairs, with scientific publications connected to those contracts. In order to do so, we developed a Python script that queries the WoS API. The script searches for the relevant procurement contract and grant identifiers in the acknowledgment section of the scientific publications in the WoS database, published between year 2000 and 2015. The Supporting Information (S1 File) provides extensive information on the data collection process and data coverage.

## Results

**Fig 1:**
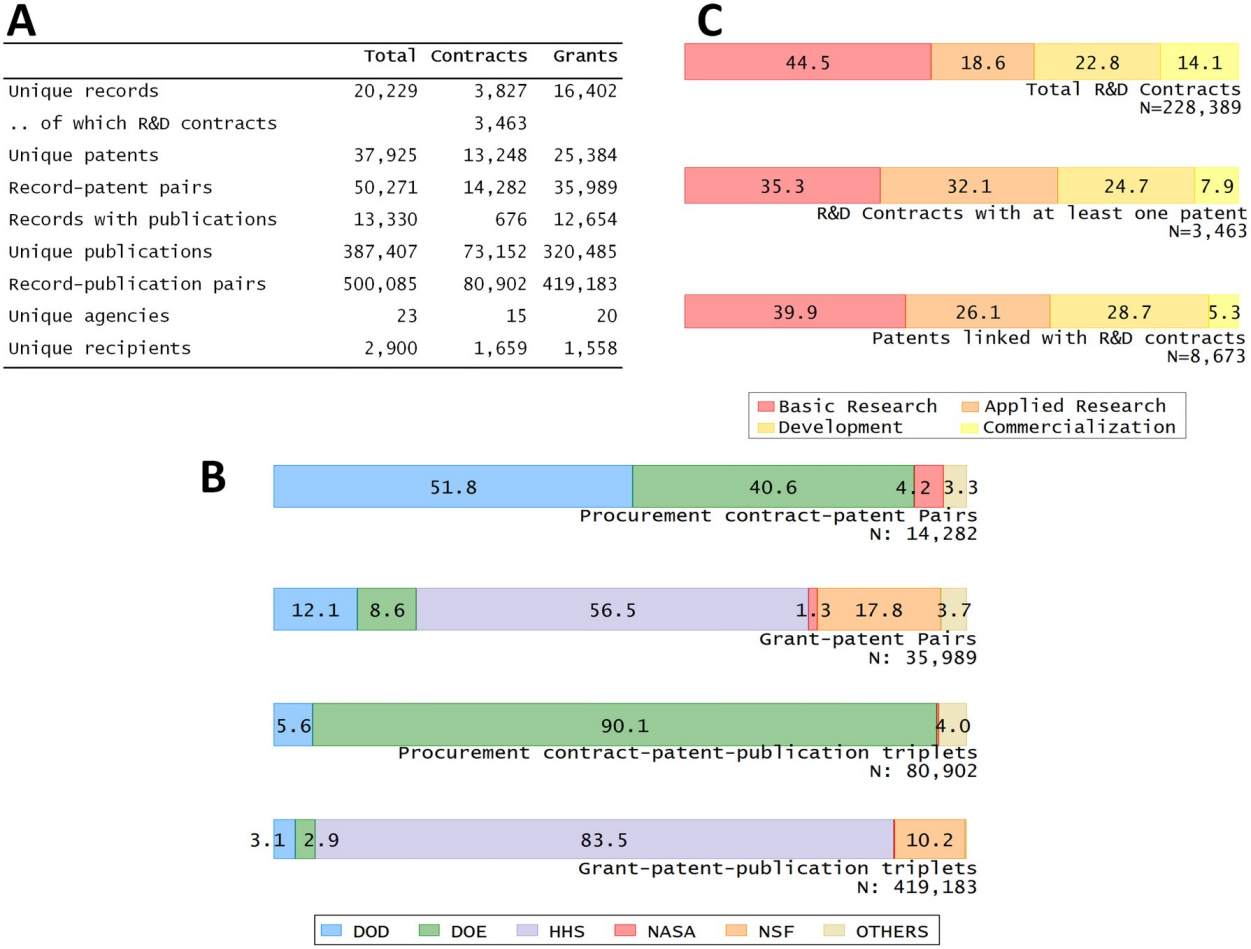
Panel A provides key descriptive statistics about the 3PFL database. Panel B displays the distribution by agency of patents and scientific publications associated with contracts and grants. Panel C reports the distribution of R&D contracts and patents by R&D stage.

Fig 1 presents an overview of the database. Panel A provides key descriptive statistics. The database contains 20,229 ‘records,’ defined as either a procurement contract (hereinafter a ‘contract’) or a research grant. By design, all these records are associated with at least one patent.

About 80 percent of records correspond to grants. The vast majority (90 percent) of contracts that lead to a patent are R&D contracts. Associated with these records are almost 38,000 patents, with about two-thirds coming from grants. To put this figure in perspective, this represents eight to nine business days’ share of the annual patents assigned to all U.S. entities. Patents can acknowledge funding from different sources and a funding source can be associated with more than one patent. Overall, there are about 50,000 such record-patent pairs. Among all these records associated with a patent, 13,330 records were also associated with a scientific publication, and these records are typically grants. There is a total of 387,407 publications for about half a million record-publication pairs. Finally, funding arises from 23 different agencies and is delivered to 2,900 recipients.

Fig 1B provides a breakdown of patent and publication numbers by agency. Patents arising from contracts are mainly associated with the Department of Defense (DOD) and the Department of Energy (DOE). By contrast, patents arising from grants are mainly associated with the Department of Health and Human Services (HHS). Regarding publications, the overwhelming majority of publications connected to contracts in the database come from the DOE whereas HHS accounts for the majority of publications connected to grants.

Because previous research in the area has overwhelmingly focused on research grants, the remainder of this paper focuses primarily on R&D contracts. Policy-makers are increasingly keen to consider innovation procurement as a technology policy tool. Public demand may actively create new markets and increase expected profits from innovation. Procurement contracts may also stimulate innovations by providing firms with the opportunity to experiment free from short-term commercial pressures [9–11]. Sometimes, public agencies have specific needs that are not met with existing products—especially concerning technologies of national security interest [12]. We know little about innovation procurement, and the 3PFL database provides a unique opportunity to enrich our knowledge on these aspects. The Supporting Information (S2 File) provides a systematic comparison between procurement contracts and grants in the 3PFL database. To summarize the main differences, procurement contracts leading to patents are primarily awarded to private companies by the DOD, whereas grants leading to patents are primarily awarded to higher education institutions and non-profit by the HHS and the NSF.

A first order question concerns the proportion of procurement contracts that lead to a patent. Fig 1B provides information on the patent activity at the extensive and the intensive margins. There were a total of 228,389 R&D procurement contracts issued between the years 2000 and 2013; 3,463 of them have led to at least one granted patent and hence appear in the 3PFL database. While this figure corresponds to a mere 1.5 percent of all R&D contracts, contracts connected to patents account for 36 percent of overall value. Applied research contracts are proportionally more likely to lead to a patent than other types of R&D. However, conditional on being associated with a patent, basic research contracts lead to more patents than other types of R&D.

**Fig 2:**
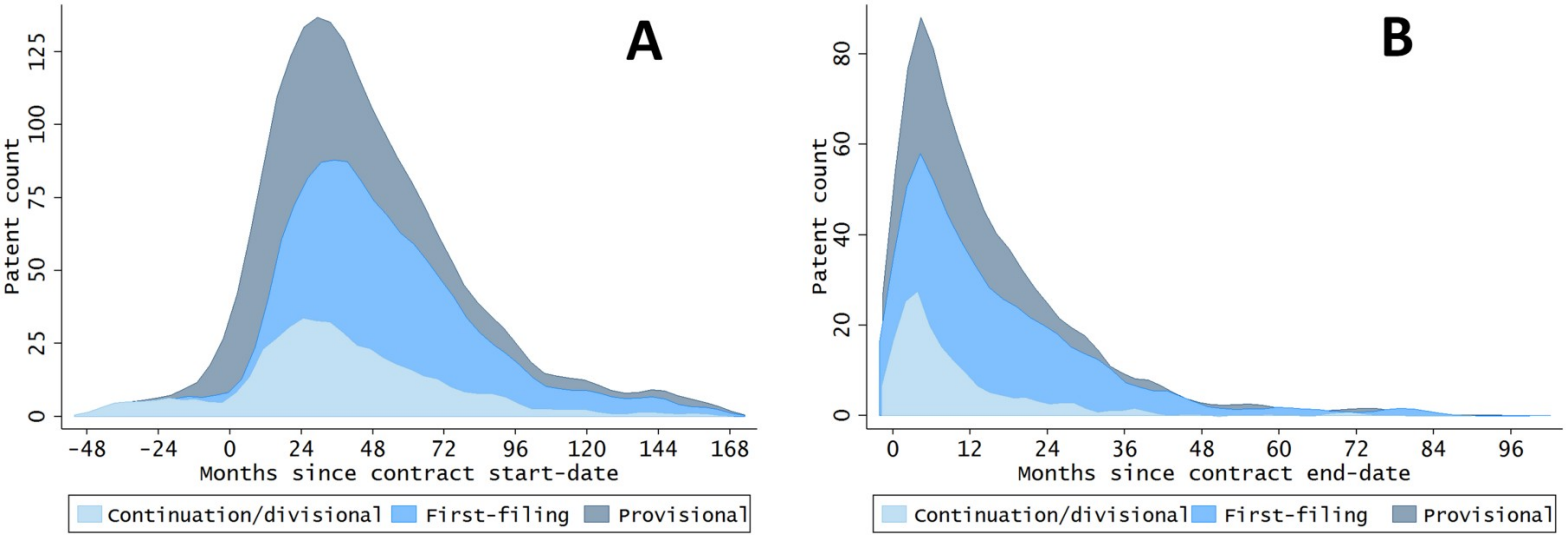
The lag is computed as the difference in months between the date of first priority of the patent application and the date of first signing of the contract (panel A) or the latest end date of the contracts (panel B). See S1 File for additional notes.

An original insight concerns the time lag between the start date of the procurement contract and the invention date (so-called R&D gestation lag). A large number of patents are filed the year of the start of the contract or in the first few years after the start of the contract (Fig 2A), thus exhibiting quite short gestation lags (33 months on average). However, such short R&D gestation lags are not peculiar to R&D procurement projects [13]. Other patents seem to have a surprisingly long gestation lag; a sizeable number of patents are still filed five years after the start of the contract. However, the majority of these patents arise from DOE contracts covering the running of national labs. In other cases, these patents are associated with renewed contracts and acknowledge the contribution of all contracts in the lineage. Interestingly, more than 1500 patents are filed after the expiration of the contract (Fig 2B). This suggests that there is an opportunity with these data to explore the extent and nature of follow-on innovation undertaken by the contracting entities.

A priori, we would expect patents related to basic research to have longer gestation lags than applied research, and a fortiori development research. To test this hypothesis we run an exploratory multivariate regression analysis of the determinants of gestation lag. The analysis is conducted at the patent-contract pair level and focuses on the effect played by the stage of the R&D work for which a given contract was awarded. The information about the stage of a R&D contract is codified in the FPDS data. The *contract-patent lag* is our dependent variable and is computed as the difference in months between the date of first priority of the patent application and the date of first signing of the public procurement contract. Our main variables of interest in the regression are *applied_research*, *development*, *commercialization*. These binary variables capture the four categories of the R&D stage to which a contract could belong. The category *basic_research* is omitted from the regression and serves as the reference group. We also control for several contract- and patent-specific characteristics, such as the size and the total length of a contract, whether it was awarded as part of the Small Business Innovation Research (SBIR) program, and for the scope of the patented invention measured as the number of independent claims. As Table 1 shows, the R&D stages have surprisingly no significant impact on the gestation-lag of a patented invention. Interestingly, SBIR contracts are associated with substantially shorter gestation-lags.

**Table 1:** Contract-patent lag and R&D stages.

	contract-patent lag				
	(1)	(2)	(3)	(4)	(5)
applied_research	0.161	1.076	-1.460	-1.320	-1.391
	(1.809)	(1.530)	(1.140)	(1.137)	(1.098)
development	14.358***	3.859*	-0.186	-0.084	-0.957
	(4.212)	(2.010)	(1.284)	(1.272)	(1.307)
commercialization	-1.493	2.721	1.274	1.307	1.068
	(3.179)	(2.392)	(2.627)	(2.638)	(2.635)
contract-length		0.319***	0.225***	0.224***	0.216***
		(0.035)	(0.026)	(0.026)	(0.025)
log_amount($)		-0.246	1.144***	1.148***	1.110***
		(0.676)	(0.258)	(0.258)	(0.265)
sbir		-4.790***	-3.852***	-3.790***	-3.711***
		(0.933)	(0.972)	(0.967)	(0.967)
competed		-6.403**	-0.922	-0.922	-0.896
		(3.094)	(1.861)	(1.838)	(1.824)
fixed-price		-0.420	0.497	0.531	-0.102
		(1.216)	(1.093)	(1.092)	(1.069)
nr_independent_claims				-0.537***	-0.665***
				(0.153)	(0.147)
contract_start-year	No	No	Yes	Yes	Yes
contract_start-month	No	No	Yes	Yes	Yes
office	No	No	Yes	Yes	Yes
R&D_category	No	No	Yes	Yes	Yes
patent_class	No	No	No	No	Yes
constant	28.314***	19.705**	51.675***	53.261***	37.676***
	(1.294)	(9.348)	(10.667)	(10.459)	(10.613)
Observations	6946	6946	6946	6946	6946
*R*^2^	0.059	0.326	0.381	0.382	0.435
*F*-test	4.25	1.58	0.86	0.78	0.65

Standard errors in parentheses

* *p* < 0.1,

** *p* < 0.05,

*** *p* < 0.01

The row *F*-test reports the results of a joint significance test for the R&D stage variables

The dollar *amount* of a procurement contract is computed by adding all the transactions recorded for a given contract in the time period we consider, i.e., 2000–2013.

An interesting pattern in the data is the fact that patents with shorter gestation lag seem to be more valuable, as assessed with commonly-used patent-based metrics of value such as the number of citations received by the patent [14]. Table 2 reports the results of a multivariate regression of the number of citations received by a patent in the 5-year time window from the filing date on the gestation lag. As the table shows, the contract-patent lag is negatively associated with the number of citations and this relationship is robust to controlling for several patent- and contract-specific characteristics. This result could signal decreasing returns to R&D, in the sense that the later patents in a contract seem to be less valuable than earlier ones.

**Table 2:** Patent value and contract-patent lag.

	patent value				
	(1)	(2)	(3)	(4)	(5)
contract-patent_lag	-0.028***	-0.027***	-0.032***	-0.024***	-0.026***
	(0.004)	(0.005)	(0.005)	(0.006)	(0.005)
contract_length		0.009**	0.003	0.003	0.003
		(0.004)	(0.005)	(0.005)	(0.005)
log_amount($)		-0.211***	-0.017	-0.028	-0.072
		(0.062)	(0.081)	(0.080)	(0.087)
sbir		0.016	0.184	0.177	0.066
		(0.250)	(0.269)	(0.269)	(0.270)
competed		-0.227	0.151	0.134	-0.184
		(0.292)	(0.304)	(0.299)	(0.316)
fixed-price		-0.481*	-0.160	-0.130	-0.217
		(0.253)	(0.255)	(0.259)	(0.246)
nr_independent_claims				0.193***	0.159***
				(0.045)	(0.043)
contract_start-year	No	No	Yes	Yes	Yes
office	No	No	Yes	Yes	Yes
R&D_category	No	No	Yes	Yes	Yes
R&D_stage	No	No	Yes	Yes	Yes
patent_filing-year	No	No	No	Yes	Yes
patent_class	No	No	No	No	Yes
constant	3.523***	6.540***	3.181**	-2.555	-0.235
	(0.241)	(1.006)	(1.399)	(1.670)	(2.701)
Observations	6946	6946	6946	6946	6946
*R*^2^	0.018	0.025	0.051	0.064	0.135

Standard errors in parentheses

* *p* < 0.1,

** *p* < 0.05,

*** *p* < 0.01

The dollar *amount* of a procurement contract is computed by adding all the transactions recorded for a given contract in the time period we consider, i.e., 2000–2013.

**Fig 3:**
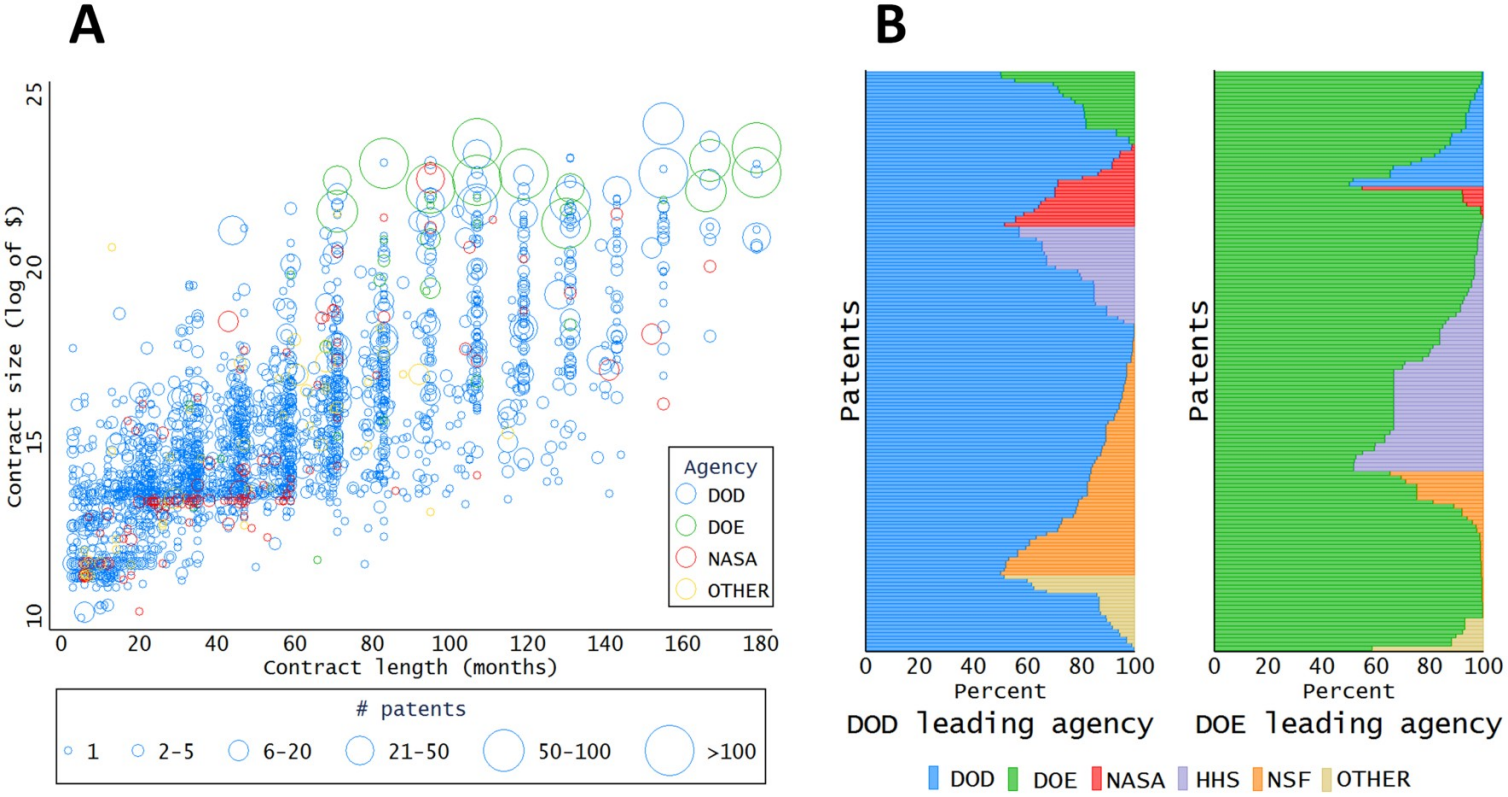
Panel B displays how much each of the two agencies involved have proportionally contributed to the development of the patent, based on the size of each contract, for contracts in which either the Department of Defense (left) or the Department of Energy (right) was the largest contributor. See SI for additional details.

The data also allow exploration of the relationship between contract characteristics and the extent of patenting. Panel A of Fig 3 shows the relationships among contract size, contract duration and patenting. Not surprisingly, contracts receiving more total funding tend to span a longer time period. However, the number of patents is only weakly correlated with both size and duration. Some contracts generate a large number of patents (more than 100 patents) but they are all around or above the half billion-dollar mark and associated with the DOE (except for one DOD contract). Table 3 reports the results from a multivariate linear regression model that suggests that, on average, a 1 percent-increase in the size of an R&D contract is associated with 0.12 percent more patents. The length of the contract has a negligible impact on the number of patents produced by a contract—thus, money matters more than time in this context. (A similar analysis run on grants provides similar elasticity estimates, see Table A in S2 File).

**Table 3:** Patents per contract and contract size.

	patents per contract		
	(1)	(2)	(3)
log_amount($)	0.125***	0.122***	0.128***
	(0.013)	(0.016)	(0.015)
contract_length	-0.002**	-0.002**	-0.002**
	(0.001)	(0.001)	(0.001)
sbir		-0.036	-0.018
		(0.024)	(0.027)
competed		0.008	-0.003
		(0.070)	(0.072)
fixed-price		0.008	0.039
		(0.032)	(0.036)
contract_start-year	No	No	Yes
office	No	No	Yes
R&D category	No	No	Yes
R&D stage	No	No	Yes
constant	-1.239***	-1.200***	-1.495***
	(0.158)	(0.225)	(0.401)
Observations	2806	2806	2806
*R*^2^	0.119	0.120	0.149

Standard errors in parentheses

* *p* < 0.1,

** *p* < 0.05,

*** *p* < 0.01

Note: The dollar *amount* of a procurement contract is computed by adding all the transactions recorded for a given contract in the time-period we consider, i.e., 2000–2013.

The *contract_length* is computed as the difference in months between the date of first signing of the R&D procurement contract and the date of latest completion in the FPDS data. We consider in the analysis only those contracts for which we could clearly tell, by the structure of the *award_id* (see S1 File), that the actual starting date is in the same year as the first transaction we observe in the data.

The data also reveal patterns of overlapping interest across agencies. It is an underappreciated fact that a sizeable share of patents is associated with contracts from more than one agency—roughly 1 in 15 patents. Panel B of Fig 3 reports the interest overlap for patents linked to contracts from two different agencies. It displays the proportional contribution of each of the agencies involved in the development of the patent, based on the size of each contract. NSF and NASA grants most often overlap with DOD funding, whereas HHS grants are mainly associated with DOE funding. Interestingly, such dual funded patents appear to be more valuable than patents funded by a single agency. Table 4 displays the results of a regression analysis and shows that patents jointly funded by two different agencies receive on average 1.02 more citations in a 5-year time window from the filing date than patents acknowledging a single funding source. This figure corresponds to a 50-percent increase in the expected citation rate.

**Table 4:** Patent value and dual funded patents.

	patent_value		
	(1)	(2)	(3)
dual_funding	0.728**	1.479***	1.012***
	(0.347)	(0.343)	(0.327)
log_cost-per-patent ($)			-0.046***
			(0.017)
nr_bwd_cites			0.047***
			(0.006)
nr_npl_cites			0.031***
			(0.007)
tot_ipc			0.056**
			(0.024)
nr_independent_claims			0.065*
			(0.038)
patent_filing-year	No	Yes	Yes
patent_class	No	Yes	Yes
constant	2.728***	4.523	1.323
	(0.076)	(2.825)	(2.809)
Observations	13248	13248	13248
*R*^2^	0.001	0.138	0.152

Standard errors in parentheses

* *p* < 0.1,

** *p* < 0.05,

*** *p* < 0.01

Given that the contract-patent relation can be a many to many relation, the variable *cost-per-patent* is computed as a fractional count based on the size of the contracts, the number of contracts associated with a patent, and the number of patents associated with a contract.

**Fig 4:**
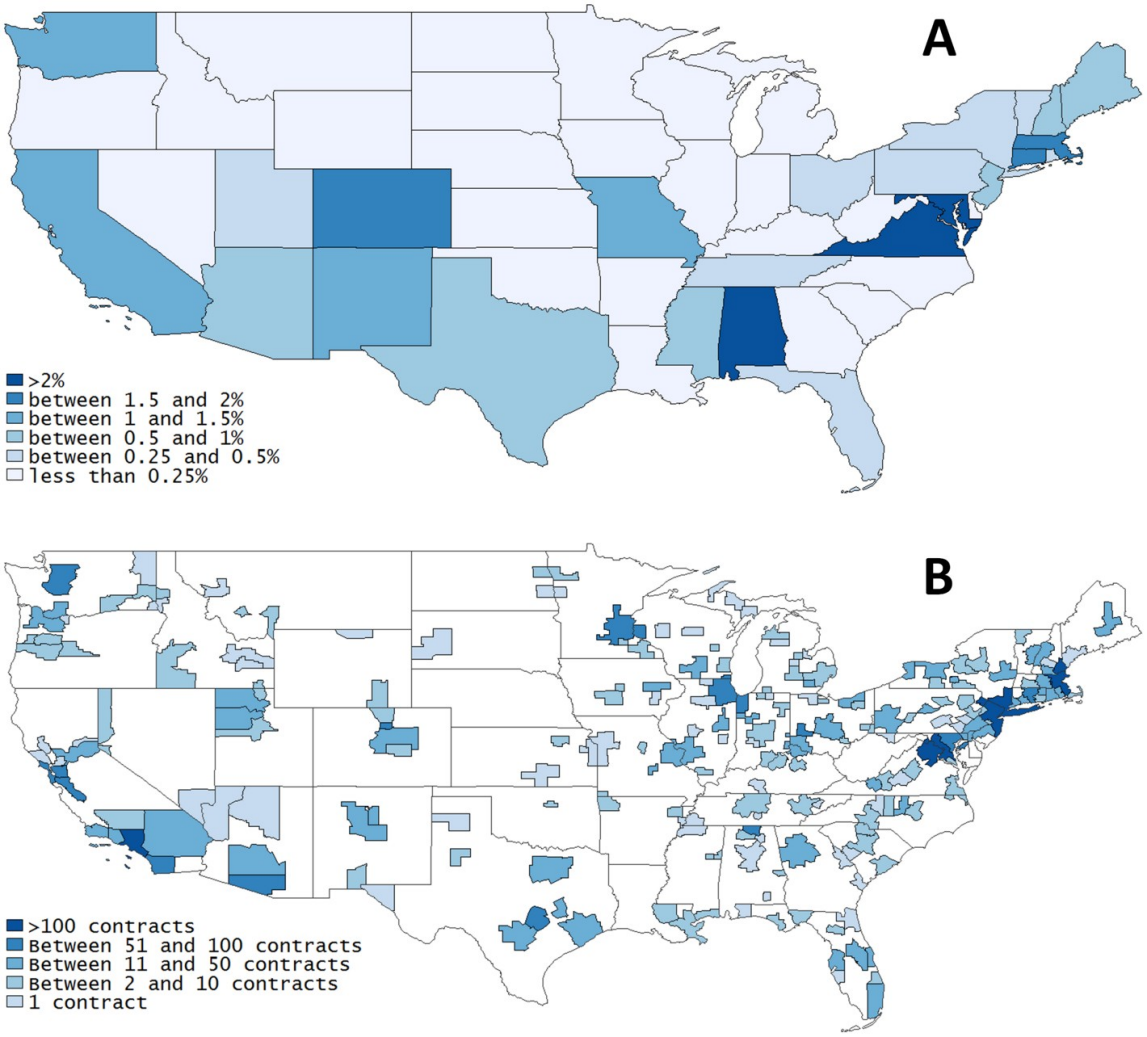
Panel A reports the distribution of the total dollar amount received for R&D procurement contracts as a percentage of GDP by state. Panel B displays the distribution of total number of R&D contracts that are associated with at least one patent by Core Based Statistical Areas.

Finally, the data can also be explored along a geographical dimension. The state of California has the most procurement contracts for the performance of R&D work, attracting more than 15 per cent of the total number of R&D contracts awarded in the study period. It is followed by Virginia (9%), Maryland (8%), Massachusetts (6.5%), and Washington, D.C (6%). As reported in Panel A of Fig 4, however, the dollar amount of R&D procurement contracts as a share of state GDP suggests a different ranking. Alabama, Virginia, Maryland, Massachusetts, and Colorado receive the largest share of R&D procurement money relative to the size of their economies. California’s share of new R&D contracts shrunk from 17.3 per cent in 2004 to 12.3 per cent in 2010, but it has the most contracts associated with at least one patent (18% of the total number of contracts linked to a patent), followed by Massachusetts (12%), New York State (5.7%), Texas (4.7%), and Virginia (4.7%). Panel B of Fig 4 shows the distribution of the total number of R&D contracts that are associated with at least one patent by Core-Based Statistical Areas. The CBSA that receives the most contracts connected to patents is the Boston-Cambridge-Quincy area, with over 400 contracts in the reference period. California has four different CBSAs among the ten largest recipients: Los Angeles-Long Beach-Santa Ana (284 contracts), San Francisco-Oakland-Fremont (99), San José-Sunnyvale-Santa Clara (99), San Diego-Carlsbad-San Marcos (87). The Washington D.C. area and the New York area attracted 190 and 166 contracts, respectively. As we mentioned, about 1.5 per cent of the R&D procurement contracts are associated with at least one patent, but some states have better performances than others in converting R&D contracts into patents. In particular Connecticut (4.3%), Minnesota (4.2%), New Hampshire (4.1%), Massachusetts (3.1%), and Arizona (3%) have a share of contracts connected to patents that is more than double the national average. California, Colorado, and New York do slightly better than average (1.8–2%), whereas Virginia, Maryland, and Washington D.C. do substantially worse than average, with a share of contracts connected to patents of 0.7, 0.5, and 0.2 per cent respectively. Geographic proximity to Capitol Hill does not seem to be a guarantee for success.

## Conclusion

This rich new database creates the opportunity for multidimensional micro-level research on the role of government research contracts and grants in facilitating the production of innovation outputs. Possible research includes topics such as the relationships between contract and contractor characteristics and innovation productivity; the quality (e.g., as measured by patent-based metrics) of innovation outputs arising from procurement contracts; and the geographic distribution of government-funded research activity and innovation outputs. Previous research has traced innovation outcomes from specific government projects, programs or institutions, but the 3PFL database offers the opportunity to understand those pieces in a broader context. Given ongoing debates about science and innovation funding, the data create a valuable opportunity to broaden and deepen our understanding of the Science of Science Policy [15].

## Supporting information

S1 File Technical appendix. The technical appendix describes the construction of the database in details.
(PDF)

S2 File Procurement contracts and grants. This appendix provides a systematic comparison between procurement contracts and grants in the 3PFL database.
(PDF)
