# Supplementary material for: The procurement of innovation by the U.S. government
Source: PLoS One. 2019 Aug 12;14(8):e0218927. doi: 10.1371/journal.pone.0218927 (PMC6690509; doi:10.1371/journal.pone.0218927)
Supplement: S1 File — The technical appendix describes the construction of the database in details. (PDF) [file pone.0218927.s001.pdf]

S1 File: Technical Appendix

Link to the original paper: *https://doi.org/10.1371/journal.pone.0218927.*

Abstract

This document is the on-line technical appendix explaining the construction of the 3PFL (Patents and Publications with a Public-Funding Linkage) database. The database links information on patented inventions and scientific publications funded by the U.S. federal government with detailed contract- and grant-level information. It combines data from multiple administrative on-line and off-line sources.

1 Introduction

This document describes the creation and the structure of the 3PFL database of federally funded patents and publications.[1] The database links information on patented inventions funded either by a public procurement contract or a research grant awarded by the U.S. federal government to detailed contract- and grant-level information, such as awarding agency, contract size and type, and to scientific publications related to the patent-contract pairs.[2] The construction of the database involves combining administrative data from multiple sources: the United States Patent and Trademark Office bulk database (USPTO-bulk), the Federal Procurement Database System (FPDS), Award Submission Portal (ASP), the European Patent Office's worldwide statistical database (PATSTAT), and the Web of Science database.

The document is structured as follows. Section 2 presents an overview of the database, its sources and structure. Section 3 describes the specific aspects of the U.S. policy and legal environment that we exploit for the creation of the database. Section 4 illustrates the different steps of the database construction. Section 5 presents the content of the database in detail. Section 6 discusses access rules.

2 Structure of the database

The database is composed of nine different tables with a relational structure. Figure A displays the logical model of the database. The `Patent_contract` table is the central table that links a patent document to grant or procurement contract identifier(s). Because grants are also contracts in the legal sense, we use the term 'contract' in a generic way to refer both to grants and procurement contracts. We will use the term 'procurement contract' to refer explicitly to a procurement contract. There is a many-to-many relationship between patents and contracts. Section 4 describes in detail the process and the methodology we used to populate this table.

Tables on the left-hand side of the model report information on procurement contracts as recovered from `USAspending.gov`. In particular, table `Procurement_information` reports time-invariant information about the procurement contract such as the funding agency, the type of contract used, the extent to which it was competed, and a description of the purchased good or service. Because a contract could run for several years and may generate several transactions, the

[1] The present document relates to version 1.0 of the database, which can be freely downloaded from the project website at `http://www.3PFL.io` or from Zenodo.

[2] Thus, publications are linked to procurement contracts only if these contracts and grants are associated with a patent.

table Procurement_year reports the amount of obligated dollars on a given procurement contract per fiscal year. The table Vendor_information reports data on contractors.

Tables on the right-hand side of Figure A display information on research grants and on the grant receivers, as retrieved from USAspending.gov. The data are stored in three different tables reporting time-invariant grant-level information (Grant_information table), year- and grant-specific information (Grant_year table), and information on the organization that received the grant (Grantee_information table).

The table patent_information displays detailed patent-level information recovered from the PATSTAT database and from the full text of the patent document as recovered from the USPTO-bulk database.

The table Paper_information reports the identifier and the year of publication of scientific publications that are related to contract-patent pairs. We recover this information by searching for contract and grant identification numbers in the acknowledgement field available in the Web of Science database.

Section 5 describes in detail the content of each of these tables.

Fig A. DATA STRUCTURE

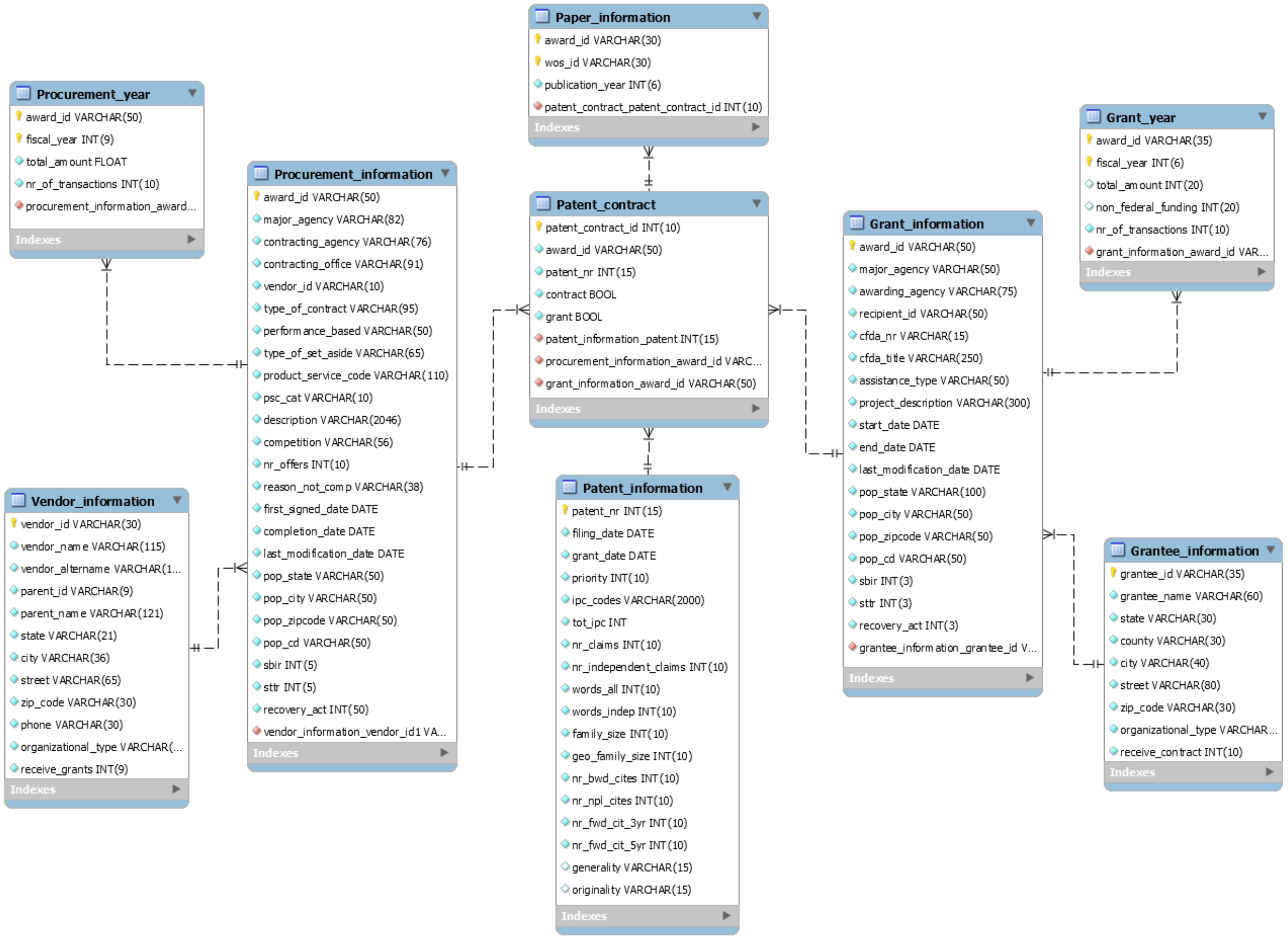

Note: Schema for version 1.0 of the 3PFL database.

3 Background and construction of the database

The main challenge of this project is to unambiguously identify patented inventions (and the associated publications) that were funded by federal money.

3.1 The Federal Acquisition Regulation and the Government interest statement

As far as procurement contracts are concerned, we take advantage of the U.S. Federal Acquisition Regulation (FAR). The FAR regulates the acquisition process of goods and services by the Federal government. Subpart 27.3 of the FAR governs patent rights under Government contracts and states that each contractor may elect to retain title to any invention made, or first reduced to practice, in the performance of work under a Government contract or subcontract for experimental, developmental, or research work.[3] In order to retain title to an invention, the contractor should disclose to the government the discovery of a patentable invention and file a patent application within a reasonable time. When the contractor chooses to take title of the patentable invention FAR 27.302 imposes that the Government retains a non-exclusive, irrevocable, paid-up license to use the invention, or to have someone else using the invention on its behalf. To ensure that the government retains a non-exclusive, royalty-free license, the FAR requires the contractor to include in the U.S. patent document a statement that acknowledges that the invention was made with Government support and identifies the governmental agency and the contract number. If a contractor fails to disclose or elect ownership within the times specified in the FAR, or fails to give a free license to the government, the funding agency may take title of the invention. The existence of these requirements in the FAR entails that it is possible to identify the patented inventions produced in the performance of work under a government contract; one simply needs to look for U.S. patents that report a government interest statement. Figure B provides an example of a typical government interest statement included in a patent document. As the figure shows, the assignee not only reports that the invention was supported by a given governmental agency but it also refers to the specific contract number(s) related to that patent application.

3.2 The Bayh-Dole Act and research grants

Although federal research grants are legal contracts between a federal agency and the recipient, they are not procurement instruments [2].[4] The FAR requirements discussed in the previous section do not apply to these kinds of contracts, generally defined as 'funding agreements'. Nevertheless, funding agreements are covered by the Bayh-Dole Act. Section 202(c) of the Bayh-Dole Act imposes requirements similar to FAR for recipients of federally funded research grants that elect to retain title to associated patentable inventions [3]. The grantee seeking patent protection for such inventions mentions the grant number and the agency that issued the grant in the government interest statement. One can then extract the information from the statement in the patent document and link patent-level information to grant-level information.

3.3 The Federal Funding Accountability and Transparency Act

In September 2006, the U.S. Congress approved the Federal Funding Accountability and Transparency Act (FFATA), sponsored by Senators Coburn, Obama, Carper, and McCain. The Act requires

[3]Initially, in accordance with the Bayh-Dole Act, only small businesses and non-profit organizations could retain title of an invention realized under a government contract. In 1983 a presidential memorandum issued by Reagan extended the scope of the FAR 27.3 to large and for-profit enterprises to mitigate the reluctance of contractors to collaborate with the federal government [1].

[4]Procurement instruments should be chosen when the government purchases products or services for its own benefit. Grants should be used when the aim of the funding is to develop technologies or knowledge in the public interest (31 U.S. Code §6303).

Fig B. Example of a government interest statement in a U.S. patent

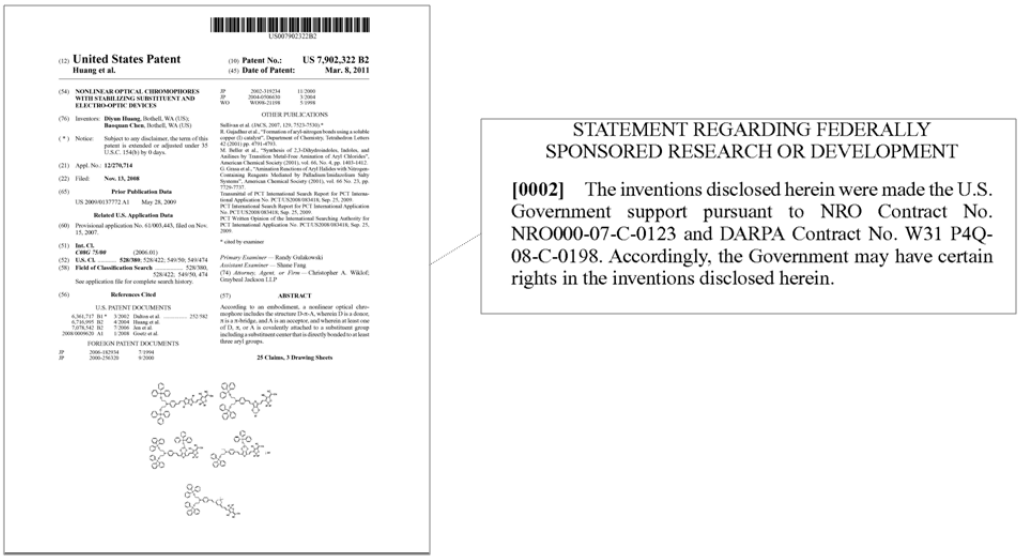

federal contract, grant, loan, and other financial assistance awards to be displayed on a searchable, publicly accessible website in order to give the American public access to information on how tax dollars are being spent. The U.S. government launched in December 2007 the `USAspending.gov` website in order to comply with the FFATA's requirements.

In relation to federal procurement contracts, the `USAspending.gov` website includes the full data from the Federal Procurement Data System (FPDS) database, from fiscal year 2000 (starting October 1999) to present.[5] The FPDS tracks every U.S. federal procurement contract whose estimated value is above $3,000, and every modification to that contract, regardless of the dollar value. The FPDS database provides several pieces of information that relate to the contract awarding phase and to the implementation stage. Particularly relevant for the construction of the 3PFL database are the data on the following: the obligated amount of the contract, the purchasing agency, the contractor, the product or service being purchased, the kind of contract (cost-plus or fixed cost), the extent to which the contract was competed, and specific governmental programs or law that provided the funds for a given contract (such as SBIR, STTR, or the American Recovery and Reinvestment Act).

Concerning grants, the `USAspending.gov` website collects data from the Award Submission Portal (ASP). The ASP is operated and managed by the Bureau of the Fiscal Service at the Department of the Treasury. Federal agencies use this platform to upload directly financial assistance files for publications on `USAspending.gov`. The system collects financial assistance transactions on $25,000+ federal awards starting from Fiscal Year 2000 that required an application process and that relate to the performance of services that are not rendered to the U.S. federal government (i.e., grants). We collect the following data: the total amount of the grant, the amount funded through non-federal matching funds (if any), the awarding agency, the awarded entity, a brief description of the implemented project, and specific governmental programs or law that provided the funds for a given grant.

[5] Additional information is available at `www.fpds.gov`.

Fig C. Python script

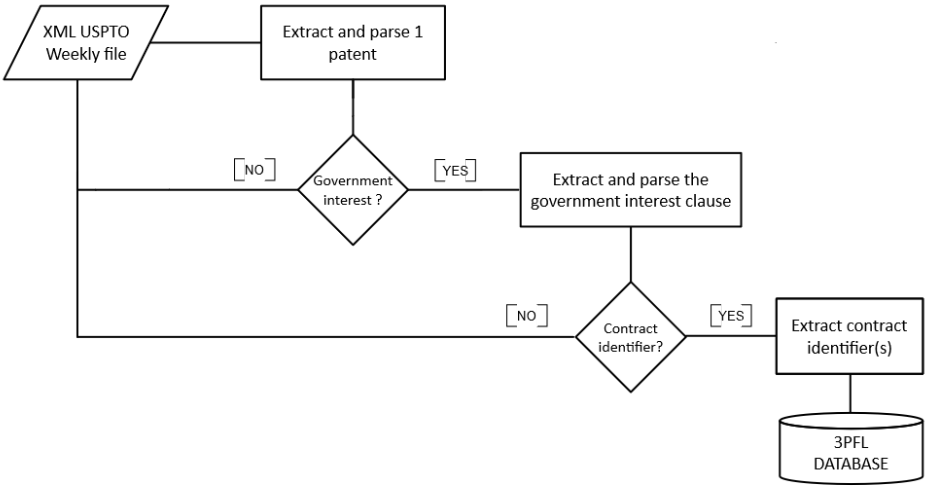

The key element for the construction of the 3PFL database is that the data available on `USAspending.gov` include a contract identification number for federal procurement contracts and a federal award identification number for grants. The contractor (or grantee) must report these identifiers in the government interest statement mentioned in the patent document (or in the funding acknowledgement for scientific publications). The FAR and FFATA requirements thus allow to clearly identify federally funded patents and to link them to the associated contract(s). The next section describes this linking in detail.

4 Database construction

The construction of the database involves four main steps. First, we identify patented inventions that contain a government interest statement. We then extract contract identifiers from the statement. Second, we match this information with the contract-level or grant-level information recovered from `USAspending.gov`. Third, we match the patent numbers with the PATSTAT-database to recover additional bibliographic information on the funded patents. Fourth, we match the contract identifiers with the WoS database, to retrieve scientific publications that are related to the contracts and the grants in the database. The present section describes each of these phases in detail.

4.1 Python Script for Parsing USPTO XML weekly files

As discussed, the main technical challenge for the database construction lies in the unambiguous identification of federally funded patents. We have developed a Python script that performs the tasks presented in Figure C.
The script operates as follows:

Stage 1. Downloading of the full text of all the patents granted by the USPTO between 2005 and 2015. The data are produced by the USPTO and are hosted by Reed Tech at `http://patents.reedtech.com`. They are stored in multiple XML files, issued weekly. The script downloads and opens 52 XML files per year for the years between 2005 and 2015 and sequentially performs the next tasks.

Fig D. Structure of the unique procurement identifier

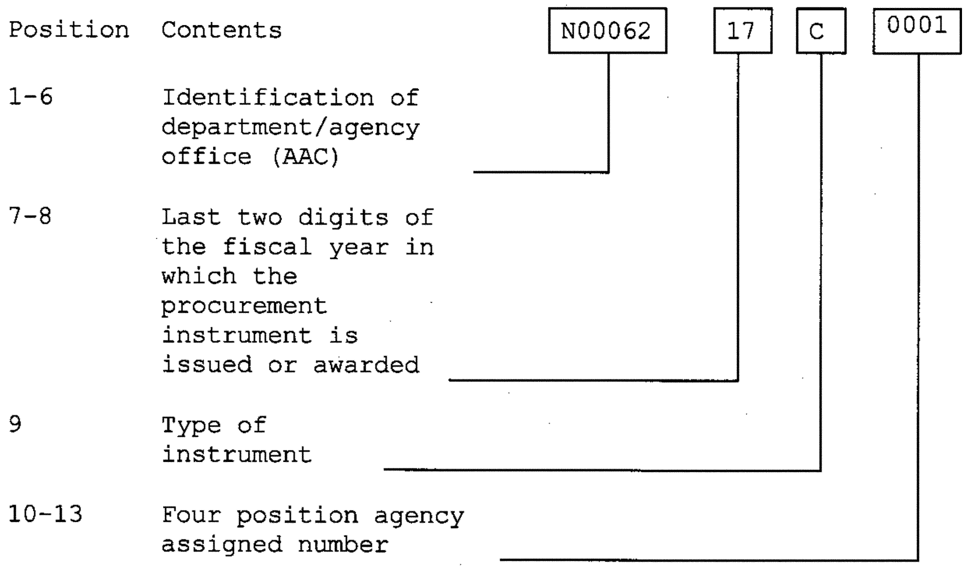

Source: `www.federalregister.gov`

Stage 2. Parsing of the content of each patent, searching for the government interest statement. In order to do that, it exploits two characteristics of the government interest statement included in the patent. First, the statement is always placed in the *description* section of the patent document. Second, although the assignee may use slightly different wording to report that a patent was realized with government support, it is possible to identify a number of keywords that allow to identify the statement. The script thus parses the *description* section of the patent looking for the presence of a set of keywords.[6] If the script finds the keywords it processes the government interest statement as described below, otherwise it proceeds to the next patent.

Stage 3. If the script identifies a patent with a government interest statement, it extracts the statement and searches for the contract identification number(s) using regular expressions. Although the structure and the length of the contract identifier(s) ultimately depend on the issuing agency, all identifiers follow a standard pattern. In the case of procurement contracts the identifier is called unique procurement identifier (PIID) and is composed of a sequence of capital letters and numbers with a specific structure (FAR Subpart 4.16). Figure D displays an example from the Department of the Navy.

As Figure D shows, the PIID is composed of four elements. The first element identifies the agency and the office issuing the contract. The next two digits account for the year in which the contract was first signed. A capital letter follows and identifies the kind of instrument used (C in the case of a contract). The last element is the contract number assigned by the agency. In the case of R&D procurement contracts, 96 per cent of the identifiers used in the period taken into account have between nine and 19 digits. In the case of grants, the identifier is called the Federal Award Identification Number (FAIN) and has a structure that is similar to the one used for procurement contracts, although the pattern for grants tends to vary more across agencies.

[6] The script first searches for the following keywords in the heading of the subsections of the patent description section: *government*, *federal*, *research*, *sponsored.* If none of the above is found, the script searches for the following expressions in the free text of the description section of the patent document: *government has*, *government may have*, *with support under*, *government owns rights*, *pursuant to contract*, *government support*, *performance of work under.*

The Python script exploits specific structure and length of the PIID and FAIN using regular expression to identify contract identifiers in the government interest statement.

Stage 4. The script extracts and stores the PIID or FAIN if found. It also records the patent number and the full text of the government interest statement in the *patent_contract* table. Otherwise, the script proceeds to the next patent.

The script described above identifies 68,640 potential patent-contract (patent-grant) relations, for a total of 47,829 unique patents granted by the USPTO between 2005 and 2015. In 33 per cent of the cases the government interest statement mentioned only the word 'contract' (22,695 patent-contract pairs), in 49 per cent of the cases only the word 'grant' (33,730 pairs), in four per cent of the cases both the word 'contract' and the word 'grant', and in the remaining 14 per cent of the cases neither the word 'contract' nor 'grant.'

4.2 Match with USAspending.gov

The next step in the database construction involves matching the contract identifiers extracted by the Python script with the actual contract and grant-level information available from `USAspending.gov`.

We first proceed with the matching of potential patent-contract pairs with the FPDS database available in the contract section on `USAspending.gov`. We find 14,282 patent-contract correspondences between patents granted by the USPTO from 2005 to 2015 and procurement contracts awarded by federal agencies between fiscal year 2000 and 2013. About 90 per cent of the positive matches (12,940 patent-contract pairs) comes from exact matching between the contract identifiers in the two databases. The remaining 10 per cent of the matches required some further processing due to minor errors by patent assignees in reporting specific characters of the contract identifiers.[7]

In those cases we adopted fuzzy matching techniques and manually checked the validity of the results by comparing the patent applicant listed in the patent document with the contractor registered in the procurement database. The 14,282 identified contract-patent pairs account for 13,248 unique patents, and 3,827 unique contracts. Indeed, a given contract may be linked to more that one patent and a given patent may be linked to more than one contract.

We then perform the same operation with the grant data available from `USAspending.gov`. In this case, we found 35,989 patent-contract correspondences, for a total of 25,384 unique patents, and 16,402 unique grants.

Overall we identified 50,271 patent-contract/grant pairs, for a total of 37,925 unique patents. This means that we correctly match about 73 per cent of the potential patent-contract pairs and 79 per cent of the unique patents identified by the Python script. A total of 18,369 of the potential patent-contract pairs are not matched. The following reasons explain why it is not always possible to find a match:

- The identifiers collected by the Python script could be false positives. The Python script is designed to collect any combination of capital letters and strings that are longer than six digits. However, a similar combination could also refer to the identification number of a specific law. For instance the text of a government interest statement may frequently refer to specific parts of the FAR, such as "48CFR 52.227-11".

- The government interest statement may refer to a contract that pre-dates the fiscal year 2000, for which no information is available on `USAspending.gov`. This is especially the case for patents granted in the earlier years. Figure E reports the distribution of matched and non-matched patents by filing year. The figure shows that non-matched patents are more frequently found in the early years. This pattern seems to confirm the possibility that the non-matched patents may frequently report the identifier of a contract that ended before fiscal year 2000.

[7] For instance, in several cases the applicants reported an identifier like 'DEAC-05-00-0R' instead of 'DEAC05-00-OR', using a zero instead of an 'O'.

Fig E. Distribution of patents related to contracts by filing year

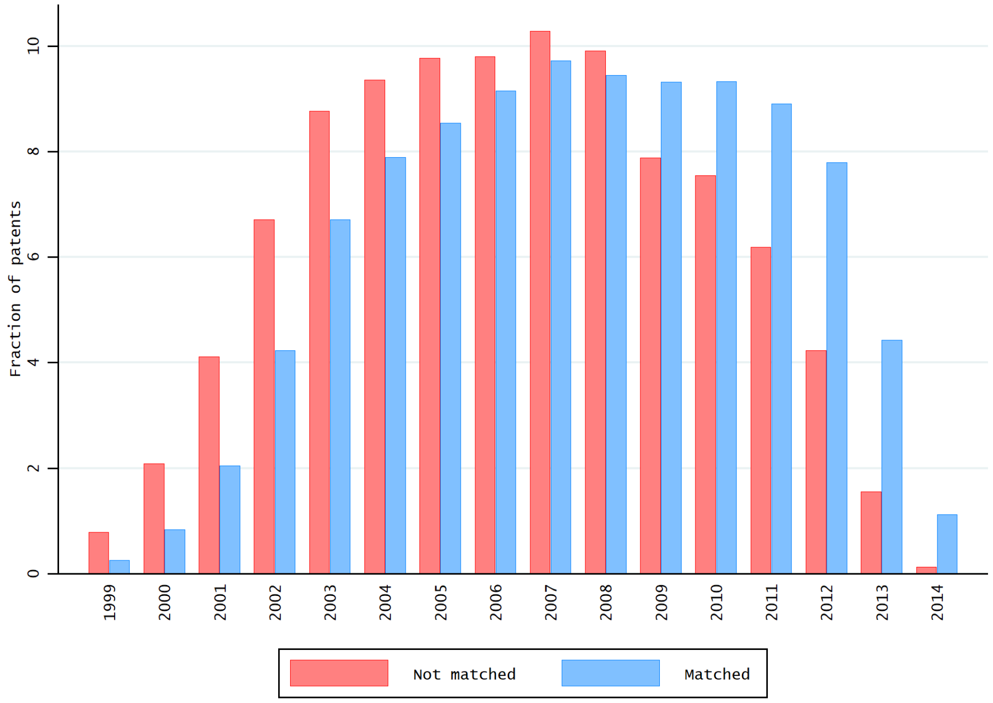

- The government interest statement may refer to contracts issued by departments that do not communicate contract information to the FPDS system (and thus, to `USAspending.gov`), or started reporting it only after a given year. For instance FPDS does not include data from selected executive agencies such as the Central Intelligence Agency and the National Security Agency.[8]

- The government interest statement may refer to a subcontract and not to a contract. Subcontract data is available only from 2010 onwards, it is hard to assess its reliability, and thus subcontracts are not included in the 3PFL database.

- The government interest statement may report a contract identifier that is not correct.

Clearly, the first issue does not pose a problem to the validity of the data. Given the specific structure of the contract and grant identifiers, if the Python script extracts a contract identifier that is not an authentic contract identifier, we will not find a correspondence in the data from `USAspending.gov`. The other issues highlighted above may introduce some selection bias in the data and we briefly discuss them in Section 4.3. Nevertheless, a positive match between the contract identifier extracted by the Python script and the identifier in the data from the FPDS and the ASP systems, ensures that we have a true positive correspondence between a patent and a procurement contract/grant.

The contract-level and the grant-level information recovered through the matching with the `USAspending.gov` allow us to populate the `Contract_information`, `Grant_information`, `Vendor_information`, and the `Grantee_information` tables of the database.

[8]For further information see the FPDS-NG User Manual, available at `https://fpds.gov/wiki/index.php/FPDS-NG_User_Manual`.

4.3 Assessing the reasons for a lack of match using procurement contracts

We focus on federal procurement contracts to assess factors that affect the likelihood to find a match. As discussed in Section 4, the rationale for focusing on procurement contracts is that the PIID (procurement contract identifier) has a more precise and standardized structure than the FAIN (grant identifier). It is thus possible to extract valuable information even for contracts for which we did not find a correspondence in the FPDS data. This is much more challenging with grants.

Out of the 18,369 pairs that are not matched with `USAspending.gov`, only 26 per cent mention the word 'contract' in the government interest statement. About 55 per cent of them mention the word 'grant', whereas 19 per cent do not mention the word 'contract' nor the word 'grant' in the statement. Although the wording used in the statement is not a conclusive proof that a given patent is actually related to a procurement contract or to a grant, the numbers suggest that the data for contract-patent pairs might be more complete than for grant-patent pairs.[9] By limiting our attention to the non-matched pairs that mention the word 'contract' (and are thus potentially related to procurement contracts), we can get a better understanding of which of the factors described in Section 4.2 determine the lack of match with the data from `USAspending.gov`.

Overall, 4,778 of the non-matched pairs mention the word 'contract'. These non-matched pairs account for 3,010 unique contract identifiers.

- About 13 per cent of these identifiers are composed of only seven digits, the minimum length we take into account. Given that a well-structured PIID should include between 12 and 16 digits (see 3.1), these short identifiers are the most prone to include false-positives and errors.

- Seven per cent of the government interest statements related to these non-matched identifiers report the word 'subcontract' in the statement. In these cases, the identifier reported in the patent document is probably a sub-contract number that is not available in the FPDS data.

- In order to understand whether the contract signing date affects the probability of finding a positive match with `USAspending.gov` data, we consider only non-matched PIIDs that are composed of 13 characters and follow the exact structure presented in Figure D. One can easily recover key pieces of information from the 13-character string. There are 765 such contract identifiers. Exploiting the well-behaved structure of the PIIDs, we can determine the year in which a contract was first signed and the agency that awarded it.

  Figure F reports the distribution of these non-matched contracts by signing year in comparison with matched contracts with the same characteristics (i.e. a PIID composed of 13 digits). As the figure shows, about 45 per cent of the non-matched contracts were signed before fiscal year 2000, whereas only about 10 per cent of the matched contracts were signed before fiscal year 2000. This pattern seems to confirm the idea that a large share of the patent-contract pairs for which we did not find a match in the FPDS data, started and ended before fiscal year 2000, the first year for which data are available in FDPS. In addition, about 70 per cent of the non-matched PIIDs come from contracts signed before 2004. As stressed by [4], the data coverage in FPDS has significantly improved over time and has made a leap in terms of quality, especially after the 2003 modernization of FPDS. The lower quality of the data before fiscal year 2004 could explain many of the non-matched contracts.

- For about 10 per cent of these non-matched patent-contract pairs, the government interest statement mentioned the NSA as the awarding agency. As discussed above, the FPDS data does not include information on contracts awarded by agencies such as the NSA or the CIA.

- In about 20 per cent of the cases the ninth digit of the PIID is either a '1', a '2', or a '3'. These numbers suggest that the identifiers reported in these cases belong to a grant and not

[9] The most common error occurs when the applicant uses the word 'contract' in a very general way and also uses it when an invention received support through a federal grant. About 20 per cent of the patents actually matched to grants used the word 'contract' instead of the word 'grant'.

Fig F. Distribution of matched and non-matched contracts by signing year

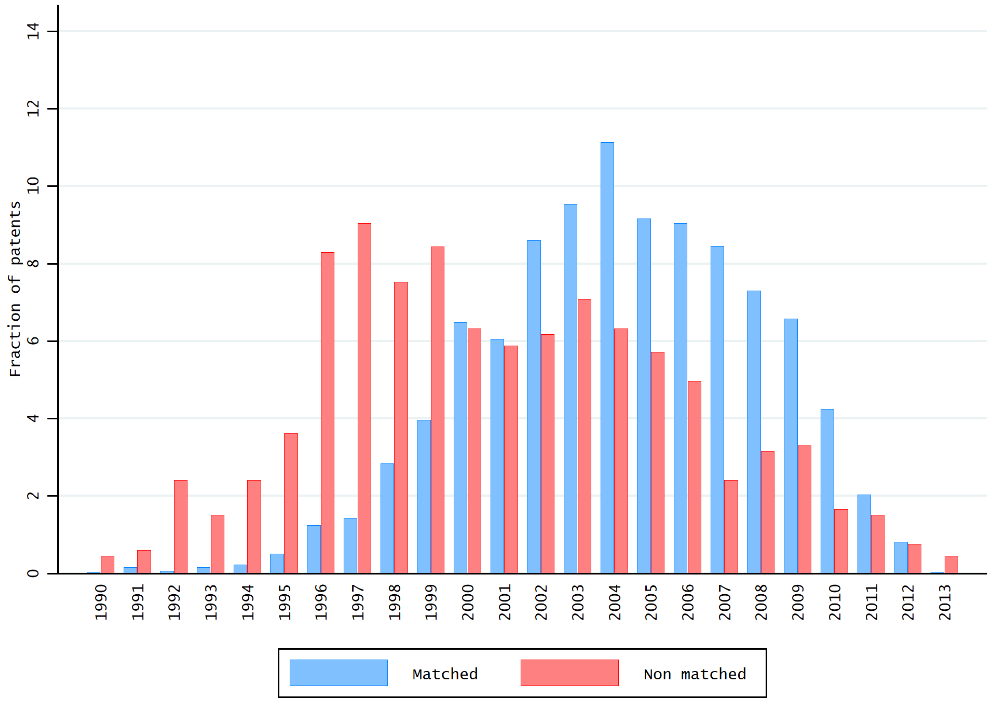

to a procurement contract.[10] In such cases, the word 'contract' is probably used in a generic way in the government interest statement.

The information presented above suggests that the matched patent-contract pairs represent a large proportion of the patent-contract pairs that could possibly be matched. If we consider all the pairs for which the government interest statement mentioned the word 'contract' and we account for the fact that about 20 per cent of the government interest statements that report the word 'contract' should instead report the word 'grant', the number of actual patent-contract pairs is probably closer to 20,000 rather than 25,000. This calculation would lead the ballpark figure of our missed patent-contract pairs to about five thousand pairs. As discussed above, almost half of the non-matched pairs are explained by the fact that the identified patents are linked to contracts awarded before fiscal year 2000, that are not available in the FPDS system, and to a lesser extent to contracts awarded between 2000 and 2003, a period for which the information on FPDS is not necessarily complete.

In addition, we still have to take into account false positives, errors in reporting, and subcontracts. Quantifying the importance of each of these factors is quite hard, but they probably account for a non-negligible amount of missed patent-contract pairs. At least in the case of procurement contracts, the matched pairs seem to represent a large share of the actual number of patent-contract pairs, most likely between 80 and 90 per cent of the total number of pairs.

Conversely, a clear assessment of the matching quality for grants is not possible as the grant identifiers (FAIN) do not have a clearly defined structure. However, for most of the non-matched pairs (about 56 per cent), the government interest statement in the patent document reported the word 'grant'. This fact seems to confirm that the data quality for the grants is lower than for procurement contracts. A corroboration to this hypothesis comes from a Memorandum of the Office of Management and Budget of the White House of June 2013, whose main objective was to improve

[10] https://www.fpds.gov/downloads/FAADS/Grants_Data_Dictionary_(Draft).pdf

data quality for USAspending.gov, especially for financial assistance. The memorandum required the agencies *"to assign financial assistance award identification numbers [FAIN] unique within the Federal agency and identify and implement a process to compare and validate USAspending. gov funding information with data in the agency's financial system"*.[11] The memorandum thus provides some evidence that, at least before 2013, federal agencies did not have a fully harmonized system to report financial assistance transactions, a fact that may explain the lower quality of the grant data.

4.4 Match with the PATSTAT database

Once the 50,271 patent-contract pairs have been matched with data from USAspending.gov we proceed to match them with the PATSTAT database.[12] PATSTAT is one of the largest patent databases available and includes abundant bibliographic information for patent applications filed in more than 100 patent offices [5].

We match our patent-contract pairs to PATSTAT based on the publication number of the granted patent document, which we recover from the USPTO full-text database. We are able to match all the patent-contract pairs with the PATSTAT database. The information recovered through PATSTAT allows populating the *Patent_information* table of the database.

4.5 Match with the Web of Science database

Finally, by exploiting Clarivate's Web of Science (WoS) database, we match the contracts from the identified patent-contract pairs, with scientific publications related to those contracts. In order to do so, we developed a Python script that queries the WoS API and searches for the relevant procurement contract and grant identifiers in the acknowledgment section of the scientific publications in the WoS database, published between year 2000 and 2015. The script recovered 500,085 contract-publication pairs, for a total of 387,407 unique publications. About 16.2 per cent of the procurement contracts related to patents are also related to at least one scientific publication. By contrast, 76.2 per cent of the grants related to patents are linked to at least one publication. The information recovered through the WoS database is then used to populate the `Paper_information` table.

5 Database description

As mentioned in Section 1, the 3PFL database is composed of nine different tables. This section describes the content of each of these tables.

5.1 The Patent_contract table

The `Patent_contract` table is the central table of the 3PFL database and it is fed by the information recovered by the Python script described in Section 3.1. The table includes the following variables:

- `patent_contract_id`: the primary key that uniquely identifies a patent-contract pair.
- `award_id`: identifies the procurement contract or grant identifier as reported on USASpending.gov. In the case of federal procurement contracts the `award_id` corresponds to the PIID, whereas in the case of grants it corresponds to the FAIN (see Section 4.2).

[11] https://obamawhitehouse.archives.gov/sites/default/files/omb/financial/memos/improving-data-quality-for-usaspending-gov.pdf

[12] For a description of the content of the database see the PATSTAT data catalog available at: https://www.epo.org/searching-for-patents/business/patstat.

- patent_nr: the publication number of the USPTO granted patent that is linked to a procurement contract or to a grant.
- contract: takes the value 1 if the award_id refers to a federal procurement contract and 0 if it refers to a grant.
- grant: takes the value 1 if the award_id refers to a federal grant and 0 if it refers to a contract.

5.2 The Procurement_information table

The Procurement_information table reports time-invariant characteristics of federal procurement contracts, sourced from USAspending.gov.[13]

- award_id: the contract identifier, the primary key of the table.
- major_agency: the major federal organization that has awarded the contract. Most common agencies in the data are the Department of Defense (DoD), the National Aeronautics and Space Administration (NASA), and the Department of Energy (DoE).
- contracting_agency: the governmental agency or bureau that executed or is otherwise responsible for the transaction. For instance, when the the major agency is the DoD, the field indicates whether the contract was awarded by the the Department of the Navy, the Army, Air force, or other smaller agencies that form part of the DoD.
- contracting_office: reports the contracting office that executes the transaction. It is a lower-level measure than the contracting agency. For example within the NASA, there are 15 different contracting offices that execute procurement contracts related to patents.
- vendor_id: identifies the contractor that won the procurement contract by means of the DUNS number.
- type_of_contract: defines the type of contract used as defined by FAR (Part 16). The contract types are grouped into two broad categories: fixed-price contracts and cost-reimbursement contracts. The contract types range from firm-fixed-price, in which the contractor has full responsibility for the performance costs, to cost-plus-fixed-fee, in which the contractor has minimal responsibility for the performance costs and the negotiated fee (i.e., profit) is fixed. In between these two extremes are different contracting schemes. The type of contract is usually decided based on the uncertainties involved in contract performance.[14]
- performance_based: is a binary variable that indicates whether the contract is a Performance-Based Service Acquisition (PBSA) as defined by FAR 37.601. A PBSA describes the requirements for the contractor in terms of measurable performance standards (i.e. quantity, quality, timeliness) as well as the method used to assess the contractor performance against performance standards.
- type_of_set_aside: reports the type of set asides (if any) determined for the contract action. Set-aside are contracts that are targeted to firms with specific characteristics. The most common type of set-aside in the data is related to small businesses.

[13]For a full description of the data available on USAspending.gov see the data dictionary available at: https://www.usaspending.gov/about/Pages/TheData.aspx

[14]For a more detailed definition of contract types and rules for selection, see FAR 16.1 https://www.acquisition.gov/far/html

- `product_service_code` (PSC): is a code used in the FPDS system to identify the product or the service procured. It has a four-character structure. In the case of R&D contracts the first character is always an 'A', the second character is alphabetic 'A to Z' and identifies the major category, the third character is numeric 1 to 9 to identify a subdivision of the major category, and the fourth character is numeric and may take values from 1 to 7, depending on the stage of R&D for which the contract is awarded.[15] For example the PSC code 'AJ11' identifies an R&D contract (A) in the major category of 'General science and technology' (J), in the subcategory of Physical Sciences (1), for the performance of basic research work (1).
- `PSC_cat`: indicates the first digit of the `Product_service_code` and thus the major category to which the product or service code purchased belong.
- `description`: provides a brief description of the goods or services bought.
- `competition`: reports the competitive nature of the contract. In particular, a contract could be awarded according to one of the following procedures:
  - Full and Open Competition: if the action resulted from an award pursuant to a sealed bid, a competitive proposal, a combination, or any other competitive method that did not exclude any potential supplier.
  - Not Available for Competition: if the contract is not available for competition.
  - Not Competed: if the contract is not competed.
  - Full and Open Competition after exclusion of some potential suppliers.
  - Follow On to Competed Action: if the contract is a follow on to an existing competed contract.
  - Competed under SAP: if the contract is competed under the Simplified Acquisition Procedure.[16]
- `nr_offers`: if the contract was competed, this variable reports the number of actual offers/bids received in response to the solicitation. If the contract was not competed, this variable takes the value one.
- `reason_not_comp`: if the contract was not competed this variable indicates the reasons the contract was not competed, as described by FAR (Subpart 6.3). The most frequent case in the data is when the supplies or services required by an agency are available from only one supplier, and no other type of supplies or services will satisfy the agency requirements.
- `first_signed_date`: indicates the date in which a contract was first signed. A contract may be modified after being signed. Such a modification can generate another transaction in the FPDS system with a new signing date for the same contract identifier. For clarity, this variable reports the earliest signing date available for a given contract in the data.
- `completion_date`: reports the estimated or scheduled completion date reported when the contract was first signed.
- `last_modification_date`: displays the last date available in the data in which a given contract was modified.
- `place_of_performance_*`: several variables that reports the place of performance of the award at the following levels: state, city, zip code, and congressional district.

[15] For a detailed description of the PSC code see the Product and Service Code Manual available at `https://www.acquisition.gov/PSC_Manual`

[16] Simplified Acquisition Procedures (SAP) are contracting methods designed to facilitate the procurement of goods and services. SAP do not require formal evaluation plans, submission of detailed technical/management plans with quotes or offers, establishing a competitive range, conducting discussions, and scoring offers. Their use is subject to designated dollar thresholds.

- sbir: indicates whether a contract was awarded as part of the Small Business Innovation Research (SBIR) program. The SBIR is a competitive award program that encourages domestic small businesses to engage in federal R&D that has the potential for commercialization.[17]

- sttr: it indicates whether a contract was awarded as part of the Small Business Technology Transfer (STTR) program. The STTR is a program that supports the expansion of the public/private sector partnership by funding projects in which small businesses formally collaborate with a research institution.[18]

- recovery_act: indicates whether a contract was funded through the American Recovery and Re-investment Act (ARRA), signed into law by President Obama in 2009 to stimulate recovery from the crisis.

5.3 The Procurement_year table

The FPDS system tracks federal procurement at the level of a transaction. A transaction includes the initial contract award and all amendments or modifications to that award. A specific contract may involve several transactions during the same fiscal year, and can last for more than one fiscal year. The `Procurement_year` table aggregates the dollar value of each contract at the contract-fiscal year level, and includes the following variables:

- `award_id`: the contract identifier (foreign key). It is not unique as there may be transactions in different fiscal years.

- `fiscal_year`: indicates the fiscal year in which a given contract has been active. The combination of `award_id` and `fiscal_year` is unique within this table.

- `total_amount`: reports the total amount in dollars of all the transactions registered for a given contract in a specific fiscal year.

- `nr_of_transactions`: indicates the total number of transactions registered for a contract in a specific fiscal year

5.4 The Vendor_information table

The `Vendor_information` table reports data on federal contractors that won at least one federal procurement contract related to a patented invention. It includes the following variables:

- `vendor_id`: identifies the contractor that won the procurement contract.[19] This identifier allows to match this table with the `Procurement_information` table. The `vendor_id` is based on the `DUNS_number` reported by the contractor.[20] This identifier is not unique within the table, but the combination `Vendor_id` and `Fiscal_year` is.

- `fiscal_year`: indicates the fiscal year in which a given contractor has been active.

- `vendor_name`: reports the name of the contractor. Because the name of a contractor could be reported in slightly different forms across records, we construct this variable as the most frequently used vendor name for a given `Vendor_id`.

[17]For a detailed description of the SBIR program see: `https://www.sbir.gov/about/about-sbir#sbir-program`
[18]For a detailed description of STTR program see: `https://www.sbir.gov/about/about-sttr#sttr-program`
[19]The term contractor and the term vendor are here used as synonymous.
[20]The `DUNS_number` is the Dun & Bradstreet number of the vendor. Federal agencies require contractors to register to the Data Universal Numbering System (DUNS) maintained by Dun & Bradstreet

- `vendor_altername`: reports the most frequent alternative name used by the contractor (if any).

- `parent_id`: reports the `vendor_id` of the parent company (if any) as reported in the FPDS data. If there is no parent company, this variable is set equal to the `vendor_id`.

- `parent_name`: indicates the name of the parent company (if any).

- `state`: state of the contractor address.

- `congressional_district`: congressional district of the contractor, as derived from the ZIP code in the contractor address.

- `city`: city of the contractor address.

- `street`: street of the contractor address.

- `zip_code`: zip code of the contractor address.

- `phone_nr`: phone number of the contractor as reported to FPDS.

- `organization_type`: reports the type of organization of the vendor. A contractor could be a corporation, another government agency, an international organization, a partnership, or another kind of U.S. government entity.

- `receives_grants`: flags whether a contractor is also receiving federal grants besides federal procurement contracts, in the time period covered by the data.

5.5 The Grant_information table

The `Grant_information` table includes variables reporting time-invariant information related to federal grants sourced from `USAspending.gov`. The information available for grants is less rich than for procurement contracts because the reporting requirements imposed for financial assistance are not as strict. The `Grant_information` table includes the following elements:

- `award_id`: the grant identifier, primary key of the table.

- `major_agency`: the major federal organization that is awarding the grant. Most common agencies in the data are the Department of Health and Human Services, the National Science Foundation, the Department of Defense, and the Department of Energy.

- `awarding_agency`: the governmental agency responsible for the transaction. For instance, when the the major agency is the Department of Health and Human Services, in most cases the `awarding_agency` is the National Institutes of Health (NIH).

- `grantee_id` : the grantee identifier. Although the `USAspending.gov` website does not always provide a numerical identifier for the recipient of financial assistance, we develop our own identifier based on the name of the recipient organization, the address of the recipient, and, when available, on the DUNS number provided by `USAspending.gov`.[21]

- `cfda_nr` : the numeric code that indicates the program under which this award was funded within the Catalog of Federal Domestic Assistance (CFDA).[22] For instance the code '93.859' identifies the *Biomedical Research and Research Training* program, managed by the National Institute of General Medical Sciences (NIGMS), one of the institutes of the NIH.

[21]Given that registering a DUNS number is mandatory for firms winning procurement contracts, grantees that have ever won a procurement contract before receiving the grant have a DUNS number.

[22]See: `https://www.cfda.gov/`

- cfda_title : displays the title of the program under which the award was funded, taken from the Catalog of Federal Domestic Assistance (CFDA).

- assistance_type: indicates the type of assistance provided by the award: whether it is a block grant, a project grant, or a cooperative agreement.

- project_description : provides a brief description of the project funded through the grant.

- starting_date: indicates the date on which the grant started.

- ending_date: reports the estimated or scheduled ending date of the grant.

- last_modification_date: grants can be modified or continued beyond the original ending date. This variable displays the last date available in the data (USASpending.gov) on which a given grant was modified.

- place_of_performance_*: several variables that reports the place of performance of the award at the following levels: state, city, zip code, and congressional district.

- sbir: indicates whether a grant was awarded as part of the Small Business Innovation Research (SBIR) program. In the case of financial assistance, the USASpending.gov website does not provide a variable that clearly flags the SBIR or the STTR contracts, however it is possible to recover the information on this kind of funding mechanism by parsing the text of the project description, which we did.

- sttr: indicates whether a grant was awarded as part of the Small Business Technology Transfer (STTR) program. As in the case of the SBIR program, it is possible to recover the information on this kind of funding mechanism by parsing the text of the project description, which we did.

- recovery_act: indicates whether a grant was funded through the American Recovery and Re-investment Act (ARRA).

5.6 The Grant_year table

There can be several transactions and modifications of the original grant in a fiscal-year. In addition, projects can run for more than one year. The Grant_year table aggregates the dollar value of each grant at the grant-fiscal year level, and includes the following variables:

- award_id: the foreign key that matches to records in the Patent_contract table and the Grant_information table.

- fiscal_year: indicates the fiscal year in which a given grant has been active. The combination of award_id and fiscal_year is unique within this table.

- total_amount: reports the total amount in dollars of all the transactions registered for a given grant in a specific fiscal year.

- total_amount: reports the total amount in dollars of all the transactions registered for a given grant in a specific fiscal year.

- nr_of_transactions: indicates the total number of transactions registered for a grant in a specific fiscal year.

5.7 The Grantee_information table

As mentioned above, there is less information available from USASpending.gov for grants than for federal procurement contracts due to different reporting requirements imposed by the FFATA. The table includes the following variables:

- grantee_id: identifies the organization that won the grant and is unique. If a DUNS number is provided in the data for the grantee, the grantee_id is identical to the DUNS number. If the DUNS number is not available, we generate a new grantee_id based on the name of the organization.
- grantee_name: reports the name of the recipient organization. Because the name of the same organization could be reported in slightly different versions, we construct this variable as the most frequently used recipient name for a given contractor identifier.
- state: state of the recipient organization address.
- county: county of the recipient organization, as derived from the ZIP code in the recipient address.
- city: city of the recipient organization address.
- street: street the recipient organization address.
- zip_code: zip code of the recipient organization address
- organizational_type: reports the type of organization of the recipient. A grant recipient could be a for-profit organization, a non-profit organization, another governmental agency, or an institute for higher education.
- receives_contract: flags whether a grantee is also receiving federal procurement contract(s) besides grants, in the time period covered by the data.

5.8 The Patent_information table

The Patent_information table provides information on patents related to a procurement contract, a grant, or both. It is populated with information coming from two main sources: the PATSTAT database and the XML files with the full text of patents granted by the USPTO (see Section 4). The table includes the following variables:

- patent_nr: the number that identifies the publication of the granted application at the USPTO. It is unique within this table and allows to connect the patent-level information to the Patent-contract table.
- filing_date: reports the date on which the patent application was filed at the USPTO.
- grant_date: reports the date on which the USPTO issued the patent.
- priority_filing: indicates whether the application is a priority filing or not. A priority filing is the first patent application filed to protect a given invention and it is usually filed at the patent office of the inventor's country of residence [6].
- ipc_codes: reports the different International Patent Classification (IPC) codes assigned to a patent. The IPC is a hierarchical system for the classification of patent applications according to the different technological fields to which they belong.[23]

[23]For additional information on the IPC see http://www.wipo.int/classifications/ipc/en/.

- tot_ipc: indicates the total number of IPC-main symbols assigned to a patent. Patents covering many IPC classes are supposedly more complex as they may rely on technologically distinct elements [7].
- nr_claims: displays the total number of claims included in the granted patent.
- nr_independent_claims: displays the total number of independent claims included in the granted patent. Independent claims describe the essential features of the invention. They can be used, e.g., as a proxy for the scope of the invention.
- words_per_indep_claims: reports the total number of words used in the independent claims divided by the total number of independent claims. A larger number of words per independent claim signals narrower independent claims.
- words_all: reports the total number of words used in the patent document.
- family_size: displays the INPADOC family size to which a patent belongs. The INPADOC family is composed of all the applications that share a priority directly or indirectly via a third application.
- geographic_family_size: displays the INPADOC family size to which a patent belongs. The INPADOC family is composed of all the applications that share a priority directly or indirectly via a third application.
- nr_bwd_cites: reports the number of backward citations that a patent makes to the relevant patented prior art.
- nr_npl_cites: reports the number of citations that a patent makes to the relevant non-patent literature (NPL). Non-patent literature may include citations to scientific journals and books.
- nr_fwd_cites_3y: indicates the number of citations received by the granted patent in the three years after the filing year.
- nr_fwd_cites_5y: indicates the number of citations received by the granted patent in the five years after the filing year.
- generality_index: reports the *generality index* as developed by [8]. This index exploits the spread of forward citations across technological fields (proxied by patent class) to compute a measure of technological pervasiveness. The generality index is bounded between zero and one, and a greater score indicates a more general technology.
- originality_index: reports the *originality index*, which is similar in spirit to the generality index except that it exploits the spread of backward citations across technological fields.

5.9 The Paper_information table

This table contains information on scientific papers related to federal procurement contracts and grants, coming from the WoS database and collected as described in Section 4.5. It includes the following variables:

- award_id: the foreign key that links to the procurement contracts or the grant identifier to which a given scientific publication is related.
- wos_id: identifies a specific scientific publication available in the WoS database. Because a scientific publication might be related to more than one contract, this identifier in not unique within the table. The combination of the wos_id and the award_id is unique.
- publication_year: reports the year of publication of the scientific paper.

6 Data access

The data are available under a Creative Commons Attribution 4.0 International license. The field `wos_id` is the property of Clarivate Analytics and is explicitly excluded from the present licensing agreement. The data are available at `http://www.3pfl.io/` or on the Zenodo platform. Users of the data should cite the original paper, available at: `https://doi.org/10.1371/journal.pone.0218927`.

References

1. Sharp GS. A Layman's Guide to Intellectual Property in Defense Contracts. Public Contract Law Journal. 2003; p. 99–137.
2. McEwen J, Bloch D, Gray R. Intellectual property in government contracts: protecting and enforcing IP at the state and federal level. Oxford University Press; 2009.
3. Rai A, Sampat B. Accountability in patenting of federally funded research. Nature biotechnology. 2012;30(10):953.
4. Liebman JB, Mahoney N. Do expiring budgets lead to wasteful year-end spending? Evidence from federal procurement. American Economic Review. 2017;107(11):3510–49.
5. de Rassenfosse G, Dernis H, Boedt G. An introduction to the Patstat database with example queries. Australian Economic Review. 2014;47(3):395–408.
6. de Rassenfosse G, Dernis H, Guellec D, Picci L, van Pottelsberghe B. The worldwide count of priority patents: A new indicator of inventive activity. Research Policy. 2013;42(3):720–737.
7. Harhoff D, Scherer FM, Vopel K. Citations, family size, opposition and the value of patent rights. Research policy. 2003;32(8):1343–1363.
8. Trajtenberg M, Henderson R, Jaffe A. University versus corporate patents: A window on the basicness of invention. Economics of Innovation and New Technology. 1997;5(1):19–50.
