# Supplementary material for: The procurement of innovation by the U.S. government
Source: PLoS One. 2019 Aug 12;14(8):e0218927. doi: 10.1371/journal.pone.0218927 (PMC6690509; doi:10.1371/journal.pone.0218927)
Supplement: S2 File — This appendix provides a systematic comparison between procurement contracts and grants in the 3PFL database. (PDF) [file pone.0218927.s002.pdf]

S2 File: Procurement contracts and grants

This file reports selected descriptive statistics that compare procurement contracts and research grants linked to patents.

We start with the distribution across agencies of contracts and grants related to patents. Figure A reports the agency distribution for procurement contracts and grants. The Department of Defense (DoD) accounts for the largest proportion of procurement contracts related to patents (84%). By contrast, the NIH and the NSF are the largest providers of grants related to patents with 56 and 20 per cent, respectively.

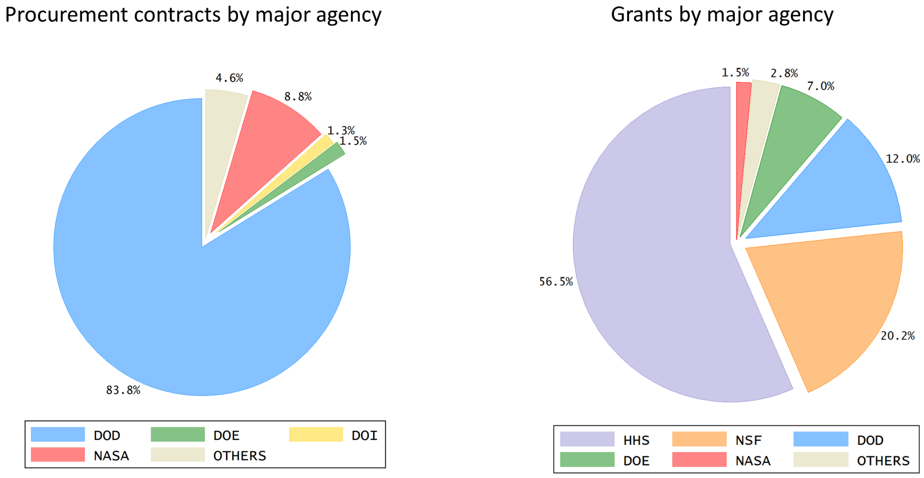

Fig A. Fraction of procurement contracts and grants related to patents by major agency

Next, we study the recipients of R&D procurement contracts and grants. Figure B illustrates that private corporations account for about nine in ten procurement contracts linked to patents and higher education institutions account for less than one in ten. The composition looks different if we consider research grants. About 84 per cent of the grants related to patents were awarded to institutes of higher education and non-profit organizations, whereas private corporations accounted for only twelve per cent of the grants.

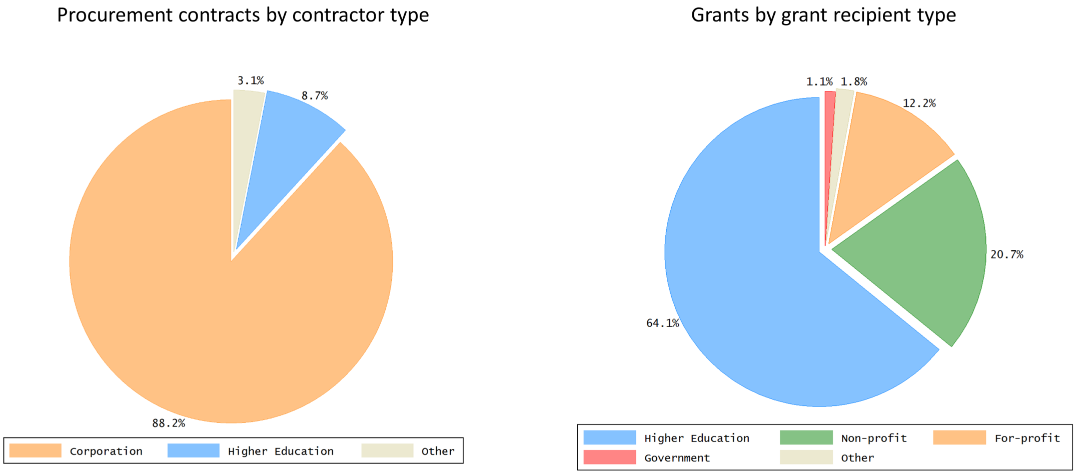

Fig B. Fraction of procurement contracts and grants related to patents by type of recipient

Procurement contracts and grants related to patents are often awarded by different agencies, operating in different technological areas, which makes a direct comparison of the two instruments difficult. To better illustrate the differences between procurement contracts and grants, we focus on the DoD, which awarded a sizable number of both procurement contracts and grants connected to patents.

Figure C reports the distribution of both instruments across the DoD sub-agencies. The distributions are strikingly similar. The main sub-agencies, namely the Department of the Army, the Air Force and the Navy, award between 85 and 90 per cent of the contracts or grants. DARPA awards three per cent of the grants connected to patents and five per cent of the procurement contracts connected to patents.

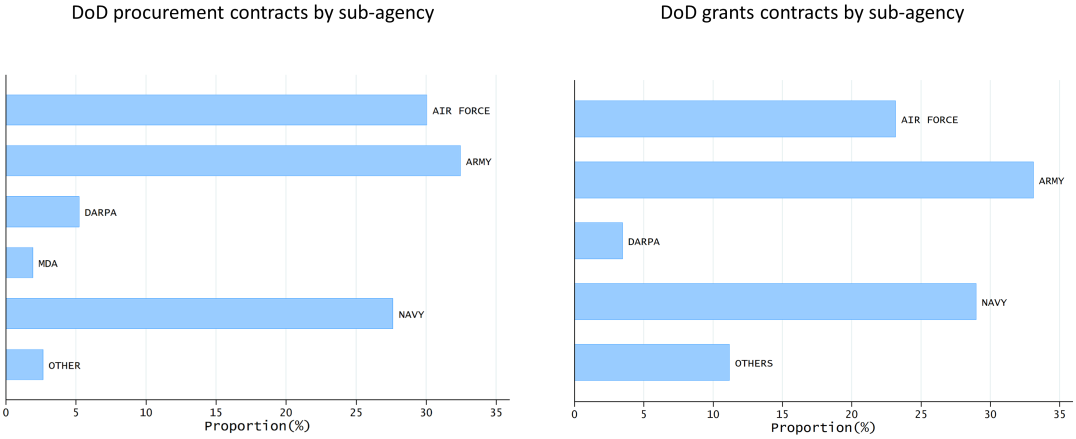

Fig C. Proportion of DoD contracts and grants connected to patents by sub-agency

The left-hand side of Figure D reports the distribution across technology field of patents related to procurement contracts and grants awarded by the DoD. Procurement-related patents are more concentrated in the Electronics and ICT sectors, whereas grant-related patents are concentrated in

the drugs and medical sector. This finding suggests that grants and procurement contracts address quite distinct research needs.

The right-hand side of Figure D depicts various indicators of patent quality. We find no significant difference between grants and procurement contracts in terms of citations to the prior art and in terms of forward citations. However, grant-related patents cite substantially more references in the non-patent literature, which are often scientific references. This fact seems to confirm the idea that grants spur innovations that are closer to the output of basic science than procurement contracts. We found little difference in terms of family size.

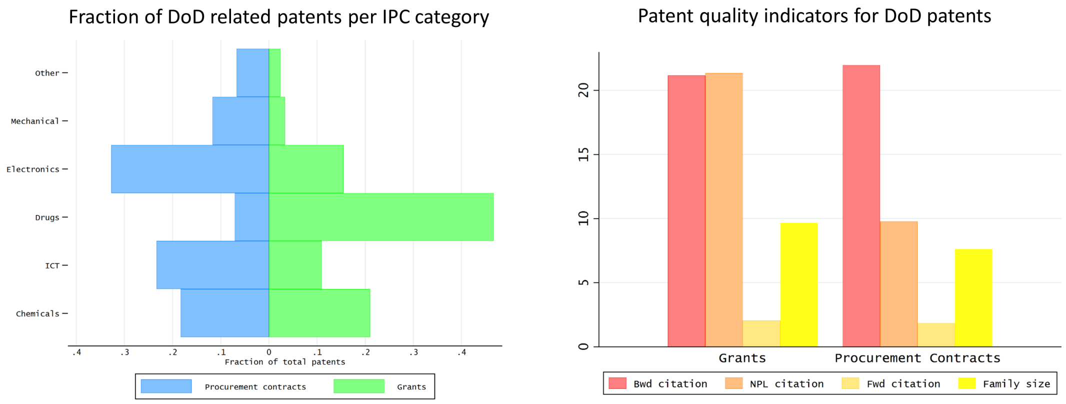

Fig D. Patent characteristics for procurement contracts vs. grants

Finally, the data on grants confirm the strong relationship between the contract size and the number of patents related to a contract that we find in the case of procurement contracts. Table A below reports the results of a regression similar to that on procurement contracts for Table 3 in the main text. It shows that a ten-percent increase in grant size leads to a one-percent increase in the number of patents that the grant generates. This effect is very similar to that obtained with procurement contracts. SBIR contracts are associated with a 9-per cent increase in the number of patents produced.

Table A. Patents per grant and grant size

	(1)	(2)	(3)
	log_patent	log_patent	log_patent
log_amount($)	0.093***	0.077***	0.105***
	(0.005)	(0.005)	(0.005)
grant_length	-0.003*	0.000	-0.003*
	(0.002)	(0.002)	(0.002)
sbir		0.023	0.093***
		(0.023)	(0.023)
agency			
grant_start			
assistance_type			
Observations	11880	11880	11880
R^2	0.052	0.059	0.113

Standard errors in parentheses

* $p < 0.1$, ** $p < 0.05$, *** $p < 0.01$

Note: The dollar *amount* of a grant is computed by adding all the transactions recorded for a given grant in the time-period we consider, i.e., 2000–2013.

The *grant_length* is computed as the difference in months between the start date of the grant and the end date reported in the USAspending data. We consider in the analysis only the grants that started after October 1, 2000, for which we observe the full funding history.
